# Does Octreoscan add value in the differential diagnosis of parapharyngeal space lesions?

**DOI:** 10.1590/0100-3984.2020.0177

**Published:** 2021

**Authors:** Raquel Baptista Dias, Alexandra Borges

**Affiliations:** 1 Radiology Department, Instituto Português de Oncologia Lisboa Francisco Gentil, Lisbon, Portugal.; 2 Radiology Department, Champalimaud Foundation, Lisbon, Portugal.

**Keywords:** Parapharyngeal space/diagnostic imaging, Receptors, somatostatin, Radionuclide imaging, Paraganglioma/diagnosis, Neurilemmoma/diagnosis

## Abstract

**Objective:**

We sought to evaluate the added value of complementary functional imaging in the differential diagnosis of parapharyngeal space lesions, as well as the benefit of performing a structured evaluation of diagnostic cross-sectional examinations.

**Materials and Methods:**

This was a retrospective study of 16 patients with parapharyngeal space lesions who were referred to our facility following a cross-sectional imaging study listing head and neck paraganglioma as a possible diagnosis. Each patient underwent somatostatin receptor scintigraphy with^111^In-pentetreotide (Octreoscan) prior to surgical resection of the lesion. In addition, the initial computed tomography (CT) or magnetic resonance imaging (MRI) scans were reviewed by two radiologists specializing in head and neck imaging, working independently, according to predefined diagnostic criteria.

**Results:**

Increased somatostatin receptor expression was observed in 14 of the 16 lesions evaluated. Histopathology of the surgical specimens showed that 11 of those 14 lesions were paragangliomas. Upon review, none of the three lesions for which there was a false-positive scintigraphy result (one intravascular meningioma and two schwannomas) were found to meet enough of the conventional imaging criteria for a diagnosis of paraganglioma.

**Conclusion:**

Structured analysis of imaging data increases the accuracy of the diagnosis of indeterminate parapharyngeal space lesions. Because of its high sensitivity, functional evaluation by somatostatin receptor scintigraphy should be considered a useful complementary tool for the detection of head and neck paraganglioma, provided that its limited specificity is taken into account.

## INTRODUCTION

Paragangliomas are neuroendocrine tumors derived from neural crest cells and can occur sporadically or as part of hereditary syndromes^**(1)**^. Genetic predisposition arises from several mutations^**(2)**^, and up to 30% of paragangliomas are hereditary^**(1,3)**^. Among the possible germline mutations, those affecting succinate dehydrogenase complex iron sulfur subunit B have been associated with greater paraganglioma aggressiveness and a higher risk of metastatic disease. Despite the fact that paragangliomas are considered benign lesions, a small proportion of the affected patients develop lymph node or distant metastases^**(4,5)**^. In addition to their capacity for dissemination to local and distant lymph nodes, paragangliomas can metastasize to the bone, lungs, and liver by hematogenous spread. The propensity for secondary spread may depend on the location of the primary tumor^**(3)**^.

Paragangliomas originate from parasympathetic tissue in the carotid body, jugulotympanic paraganglia, or vagus nerve, only rarely producing significant amounts of catecholamines (nonsecretory paragangliomas). That differentiates head and neck paragangliomas from their sympathetic lineage-derived counterparts, which almost always secrete catecholamines^**(6)**^, leading to potentially harmful cardiovascular and cerebrovascular vasoactive effects. In contrast, patients with head and neck paragangliomas are often asymptomatic until symptoms from mass effect and compression of adjacent structures ensue, most commonly lower cranial deficits, dysphagia, or otologic complaints. Tympanic paragangliomas can be detected through physical evaluation prompted by complaints of pulsatile tinnitus and through visualization of a reddish retrotympanic mass at otoscopy. However, for the differential diagnosis and evaluation of the extent of jugular, jugulotympanic, vagal, and carotid body paragangliomas, it is necessary to perform a radiological evaluation. In addition, these highly vascularized lesions may be difficult to access for diagnostic biopsies, particularly when located in the deep neck spaces, such as the parapharyngeal space.

Conventional imaging continues to be the first-line modality for the localization and precise delineation of head and neck paragangliomas. Imaging studies typically depict paragangliomas as well-defined soft-tissue masses that show intense, arterial-like enhancement after intravenous contrast administration. On magnetic resonance imaging (MRI), paragangliomas show high signal intensity in T2-weighted (T2W) sequences, with multiple serpentine and punctate areas of signal void, reflecting flow voids in larger intratumoral vessels^**(7)**^. Less often, these lesions also show focal areas of high signal intensity on T1-weighted (T1W) images and susceptibility artifacts on T2* or susceptibility-weighted imaging, indicative of petechial hemorrhages^**(8)**^. Compared with MRI, computed tomography (CT) affords better evaluation of temporal bone invasion for jugular and tympanic paragangliomas, showing a typical “moth-eaten” permeative pattern. However, it is not always possible to exclude the major differential diagnoses, including schwannomas, meningiomas, and secondary lesions, with confidence on the basis of imaging features alone. Skull base lesions can be particularly challenging in this regard. It is essential that the radiologist produce a structured report, stating an opinion regarding whether or not paraganglioma is the most likely diagnosis. Nevertheless, if a lesion does not meet enough criteria for a diagnosis of paraganglioma, then the need for further complementary evaluation should be clearly referenced in the report.

Functional nuclear imaging provides a complementary approach to conventional imaging that has long been useful for the detection of catecholamine-producing paragangliomas,^123^I- and^131^I-metaiodobenzylguanidine (^123^I/^131^I-MIBG) typically being used^**(2)**^. For the detection of head and neck paragangliomas, suitable options include agents that bind somatostatin receptors^**(9)**^, such as^111^In-pentetreotide and^68^Ga-labeled somatostatin analogue peptides as well as nonspecific agents such as ^18^F-fluorodeoxyglucose. Functional imaging with positron emission tomography (PET)/CT using gallium-labeled peptides is currently considered the gold standard for the study of head and neck paragangliomas^**(10,11)**^, although the technique has yet to become widely available. As an alternative, somatostatin receptor scintigraphy (SRS) with^111^In-pentetreotide, also known as an Octreoscan, has been shown to be very useful for the detection of parasympathetic head and neck paragangliomas^**(12,13)**^, for which it is superior to^123^I/^131^I-MIBG imaging^**(9)**^. In addition, SRS is useful for detecting metastatic disease (although not primary lesions) in patients with sympathetic paragangliomas^**(14)**^. The discrepancy in the sensitivity of this technique is thought to reflect the expression of specific somatostatin receptors by each subtype of paraganglioma, for which^111^In-pentetreotide has variable affinity.

Functional imaging methods are considered particularly useful in familial forms of paraganglioma, which are frequently multicentric, and have been recommended for detecting residual tumor persistence after surgery. This is particularly relevant given the relatively high (30%) incidence of subtotal resection of a paraganglioma^**(15)**^, which becomes more likely when the tumor involves the skull base, where complete surgical resection can result in increased patient morbidity.

The present work was designed to address two main goals: to determine whether functional imaging by whole-body SRS adds value in the investigation of patients with indeterminate parapharyngeal lesions; and to assess the usefulness of performing a structured evaluation of conventional imaging data based on the features considered “typical” of paragangliomas, attempting to determine whether this approach enables a confident hypothesis regarding the most likely diagnosis. To address the first question, we performed a retrospective evaluation of a sample of 16 patients referred to our facility after undergoing a cross-sectional imaging examination in which head and neck paraganglioma was listed as a possible diagnosis. In all cases, SRS was performed before surgical resection. We then compared the expression of somatostatin to the histopathological analysis of the surgical specimens, in order to assess the sensitivity and specificity of SRS for the diagnosis of paraganglioma. To address the second question, we performed a structured, independent, review of the cross-sectional imaging studies on which the initial referral to our facility was based.

## MATERIALS AND METHODS

We retrospectively evaluated a convenience sample of 16 patients who were referred to our facility between 2006 and 2017. Despite its limitations, a convenience sample provides an appropriate option when studying a rare disease, given that it is often challenging to attain an adequate sample size under these conditions^**(16)**^.

### SRS criteria for the diagnosis of paraganglioma

Upon referral, each patient underwent whole-body SRS, 4-6 h after intravenous administration of^111^In-pentetreotide, with acquisition of whole-body planar images in a large field-of-view gamma camera. The SRS results were deemed positive when uptake of^111^In-pentetreotide exceeded the background noise.

### Conventional imaging criteria for paraganglioma diagnosis

Images were reviewed by two radiologists with 4 and 25 years of experience, respectively, in head and neck imaging, who were blinded to the patient histories and were working independently. The imaging criteria established for a presumed diagnosis of a paraganglioma in the parapharyngeal space on CT and MRI included the following: the presence of a soft tissue mass in the post-styloid parapharyngeal space, causing ventral displacement of the internal carotid artery and jugular vein; and intense, homogeneous contrast enhancement after intravenous contrast administration, comparable to that of the internal carotid artery. Permeative mastoid bone erosion was considered an additional diagnostic imaging criteria for CT. Other diagnostic criteria for MRI included a signal from the mass that was (in comparison with the muscle) isointense or hypointense on T1W images and hyperintense on T2W images; and serpentine or punctate areas of signal void, with punctate areas of high signal intensity within the lesion, on T1W images. Whenever a dynamic contrast-enhanced MRI study was available, a short time to peak (similar to that of the internal carotid artery) was also considered diagnostic. These additional imaging features were not considered necessary for the presumed diagnosis of lesions measuring less than 1.5 cm. In SRS and conventional imaging studies alike, the presence of additional lesions suggestive of synchronous paragangliomas or metastases was also investigated.

Following SRS evaluation, patients underwent surgery at our facility. The correlation between conventional imaging results, functional imaging by SRS, and pathology data from surgical specimens was determined.

## RESULTS

### Patient sample characterization

Our sample was composed of 12 females and four males. The mean age at diagnosis was 49 years, similar to the 51.8 years reported in a recent retrospective study^**(17)**^. In our sample, there were a total of 20 lesions, with a mean size of 46 × 26 mm (largest perpendicular diameters) and individual dimensions that ranged from 17 × 11 mm to 80 × 41 mm. Additional lesions, consistent with synchronous paragangliomas, were found in three patients. A lesion suggestive of metastatic disease was found in one patient.

### Comparison between SRS results and pathology findings

**Figure 1:**
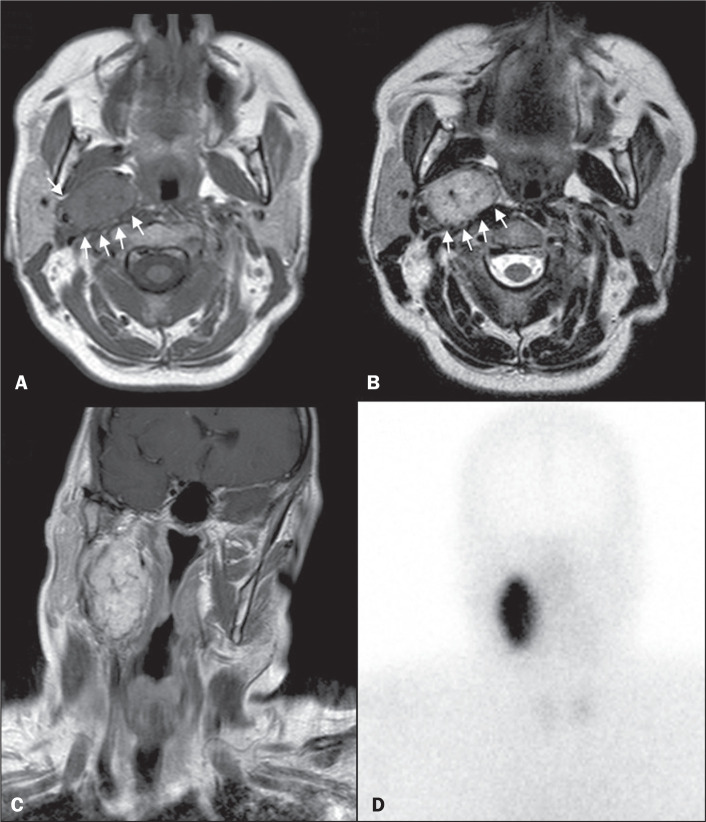
MRI and planar SRS images of patient A5, who received a confirmed diagnosis of paraganglioma. Axial T1W and T2W images (A and B, respectively) depicting a well-defined lesion (arrows), displacing the vascular bundle laterally and showing intense enhancement (C), in the right parapharyngeal space. Flow voids are part of the typical “salt and pepper” pattern. Planar SRS image (D) demonstrating strong^111^In-pentetreotide uptake.

The patients included in our sample all had lesions that were considered eligible for surgical resection. Following surgery, we analyzed data from conventional imaging, functional imaging, and surgical specimen pathology (Table 1). The SRS result was positive in 14 patients, as exemplified by the conventional and functional imaging data for patient A5 (Figure 1), and the presumptive diagnosis of paraganglioma was confirmed by pathology in 11 of those patients. Three patients (patients A8, A13, and A16) had multiple lesions, and two (patients A8 and A13) had previously undergone partial resection. In patients A8 and A13, SRS accurately detected existing residual lesions, excluding post-therapeutic changes as the most likely differential diagnosis.

**Figure 2:**
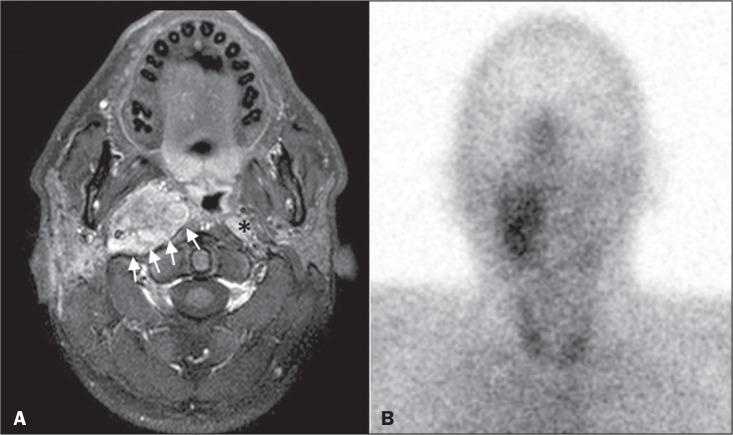
MRI and SRS images of patient A1, who received a confirmed diagnosis of metastatic paraganglioma. Axial gadolinium-enhanced fat-suppressed T1W image (A) shows a hypervascular lesion in the right parapharyngeal space (arrows) and a left retropharyngeal lymph node (asterisk). SRS (B) missed the metastatic lymph node.

In patient A1, in addition to the primary lesion, there was metastasis to the left retropharyngeal lymph node, the metastatic lesion being detected on MRI and later confirmed by pathological analysis. That lymph node, which measured 14 × 10 mm and had a short axis of approximately 1 cm, was not detected by SRS and was therefore the only false-negative result (Figure 2). In fact, the sensitivity of SRS is known to be size dependent^**(11)**^, so it is possible that this secondary lesion fell under the minimum resolution capacity of the technique.

**Figure 3:**
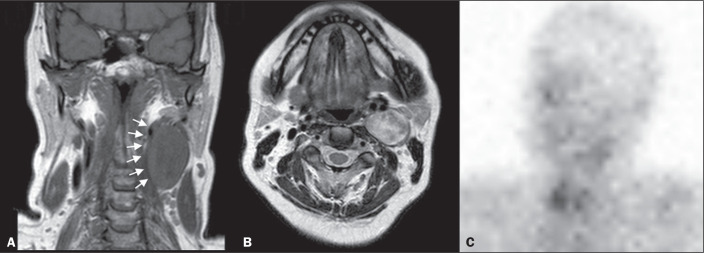
MRI scan of patient A2, in whom the SRS result was positive and the final diagnosis was schwannoma. A coronal T1W image (A) and an axial T2W image (B) show a well-defined ovoid mass (arrows in A) adjacent to the left carotid artery, heterogeneously hyperintense on T2W images, but lacking a “salt and pepper” pattern. The hypothesis of paraganglioma was supported by a positive SRS result (C).

**Figure 4:**
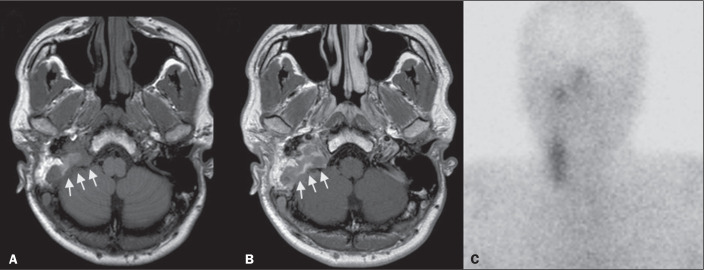
MRI and SRS images of patient A9, in whom the SRS result was positive and the final diagnosis was endovascular meningothelial meningioma. Axial unenhanced (A) and gadolinium-enhanced (B) T1W images showing a heterogeneous soft-tissue lesion (arrows) involving the right jugular foramen and the right sigmoid sinus. The lesion showed intense enhancement after gadolinium administration (B). The hypothesis of paraganglioma was supported by a positive SRS result (C).

There were three patients with false-positive SRS results (patients A2, A7, and A9), all of whom had lesions that showed increased uptake of^111^In-pentetreotide, a finding that was discordant with the subsequent pathological evaluation of the corresponding surgical specimens. In patient A7, the imaging findings were considered suggestive of parapharyngeal space schwannoma. In patients A2 and A9, the results of the initial conventional imaging evaluations were inconclusive. In both of those cases, SRS showed overexpression of somatostatin receptors (suggesting the presence of a paraganglioma). However, the pathology study revealed a vagus nerve schwannoma in patient A2 (Figure 3) and an endovascular meningioma in patient A9 (Figure 4). Therefore, in patients A2, A7, and A9, additional functional imaging was unable to disambiguate among the hypotheses of paraganglioma, schwannoma, and meningioma.

**Table 1:** Patient demographic data, lesion characteristics, and pathology results.

Patient	Age (years)	Sex	Prior history	Lesion location and size	SRS	Final pathology diagnosis
A1	29	Male		Right PPS (80 × 41 mm)	+	PG with metastatic lymph node
A2	57	Female		Left PPS (49 × 30 mm)	+	Schwannoma
A3	67	Female		Right JT (56 × 38 mm)	-	Schwannoma
A4	71	Female		Left PPS (29 × 18 mm)	+	PG
A5	58	Female	PTC	Right PPS (63 × 39 mm)	+	PG
A6	47	Female	PTC	Left PPS (25 × 18 mm)	+	PG
A7	32	Female		Right PPS (55 × 34 mm)	+	Schwannoma
A8	42	Female	Resected JT + vagus PGs	Right PPS (20 × 15 mm) + left JT (28 × 19 mm)	+/+	Multiple PGs
A9	25	Male		Right PPS with skull base involvement (142 × 22 mm)	+	Endovascular meningioma
A10	61	Female		Left JT (24 × 21 mm)	+	PG
A11	61	Female		Left PPS (49 × 28 mm)	+	PG
A12	39	Female		Left PPS (48 × 36 mm)	+	PG
A13	42	Male	Resected JT + bilateral carotid PGs	Right tympanic/PPS (22 × 22 mm) + left PPS (17 × 11 mm)	+/+/+	Multiple PGs
A14	54	Female		Right PPS (71 × 36 mm)	+	PG
A15	62	Male		Left PPS (51 × 31 mm)	-	Schwannoma
A16	35	Female		2 right PPS (54 × 26 mm; 22 × 17 mm) + 1 left PPS (23 × 16 mm)	+/+/+	Multiple PGs

PPS, parapharyngeal space; PG, paraganglioma; JT, jugulotympanic; PTC, papillary thyroid carcinoma.

**Figure 5:**
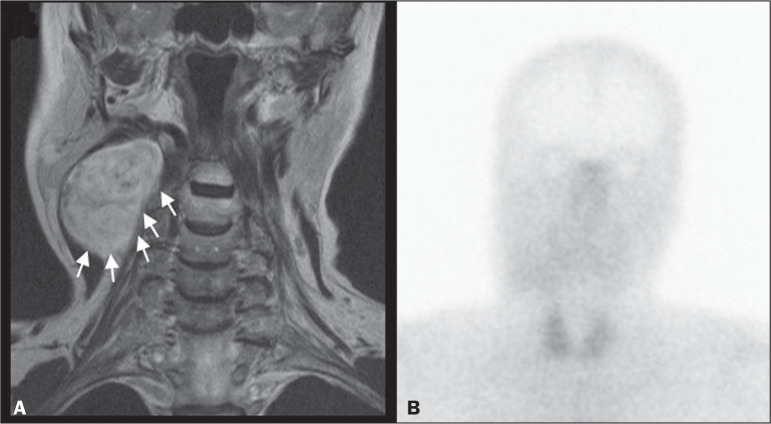
MRI and SRS images of patient A3, who received a confirmed diagnosis of schwannoma. Coronal contrast-enhanced T1W image (A) depicting a fusiform lesion (arrows) interposed between the right carotid artery and internal jugular vein, extending superiorly to the skull base. SRS did not detect somatostatin receptor overexpression (B).

The SRS produced a true-negative result in two patients (patients A3 and A15). In both of those cases, the pathological aspects of the surgical specimen were consistent with schwannoma, as exemplified in Figure 5 for patient A3.

Evaluation of patient medical histories revealed that two patients in our sample (patients A5 and A6) had previously been diagnosed with papillary thyroid carcinoma (Table 1). Both of those patients had already undergone surgical resection before being referred to our facility for investigation of parapharyngeal space lesions.

### Structured review of initial referral cross-sectional examinations

To evaluate the added benefit of having the initial imaging evaluation performed by trained radiologists, we also conducted a blinded, retrospective, structured analysis of the cross-sectional imaging studies on which the initial patient referrals were based (Table 2). Following patient randomization and diagnosis occultation, imaging data were analyzed by the two radiologists, working independently. The results were subsequently compared between the two reviewers. Identification of at least two “typical” imaging criteria was required for a lesion to be considered a paraganglioma. High (94%) interobserver agreement was achieved in deliberating whether each lesion met enough diagnostic imaging criteria to be considered a paraganglioma. Both reviewers correctly excluded the hypothesis of paraganglioma in five patients who would later receive pathological confirmation of another etiology. Hence, imaging features alone were also sufficient to exclude paraganglioma from the differential diagnosis of the three lesions that would come to produce false-positive SRS results. The level of interobserver agreement was also high (94%) in the detection of synchronous lesions and slightly lower (88%) in the evaluation of metastatic lymph node involvement.

**Table 2:** Results from independent reviews of initial cross-sectional imaging examinations.

Patient	Main diagnosis		Synchronous		lesions Metastasis		Pathology
	RR 1	RR 2	RR 1	RR 2	RR 1	RR 2	
A1	PG	PG	No	No	No	No	PG
A2	Not PG	Not PG	No	No	No	No	Schwannoma
A3	Not PG	Not PG	No	No	No	No	Schwannoma
A4	PG	PG	No	No	No	No	PG
A5	PG	PG	No	No	Yes	Yes	PG
A6	PG	PG	No	Yes	No	No	PG
A7	Not PG	Not PG	No	No	No	No	Schwannoma
A8	PG	PG	No	No	No	No	PG
A9	Not PG	Not PG	No	No	No	No	Meningioma
A10	PG	PG	No	No	Yes	Yes	PG
A11	*	PG	No	No	No	No	PG
A12	PG	PG	No	No	Yes	No	PG
A13	PG	PG	Yes	Yes	No	No	PG
A14	PG	PG	No	No	No	No	PG
A15	Not PG	Not PG	No	No	No	No	Schwannoma
A16	PG	PG	Yes	Yes	Yes	No	PG

RR, reviewing radiologist; PG, paraganglioma.

* Insufficient data.

## DISCUSSION

Conventional imaging continues to be the first-line modality for the diagnosis and precise delineation of head and neck paragangliomas, providing accurate assessment of tumor margins and invasion of adjacent structures. As we have shown here, it is essential that the evaluation of conventional imaging studies be performed by radiologists specializing in head and neck imaging. It is equally important to perform a structured analysis of the imaging data, such that the decision to include paraganglioma in the differential diagnosis of a given lesion is based on objective criteria. Using this approach, high interobserver agreement was achieved regarding whether each lesion met enough diagnostic imaging criteria to be considered a paraganglioma. Structured scrutiny of the imaging studies further enabled the exclusion of a diagnosis of paraganglioma in the three cases with false-positive SRS results. Conventional imaging is expected to perform less well in the detection of metastatic lesions, particularly distant metastases. Owing to their limited spatial scope, conventional cross-sectional studies will also miss synchronous lesions outside the compartment to which the study is directed.

One major advantage of performing complementary functional imaging is that it allows whole-body examination, thus optimizing the detection of multifocal disease. To that end, PET using^68^Ga-labeled DOTA peptides (somatostatin agonists), a recently developed functional imaging method, is currently considered the first-line imaging modality for the evaluation of cervical paragangliomas^**(11)**^. The method has a sensitivity of 100% and a false-positive rate comparable to that of SRS. However, despite the excellent results it provides, PET using^68^Ga-labeled DOTA peptides is still not widely available. It is therefore still pertinent to evaluate the added value of using alternative methods of functional imaging.

In our study, SRS showed high (92%) sensitivity for paraganglioma detection, which is in keeping with data from three previous studies with similar-sized samples^**(13,18,19)**^. Notably, sensitivity values from previous studies may be overestimated owing to sample bias, because only lesions of a considerable size are likely to have undergone further investigation and subsequent evaluation by SRS. In fact, if previous studies had included smaller lesions (< 1 cm), many of those would have presumably fallen below the minimum detection level of gamma cameras, thereby increasing the rate of false-negative results^**(9)**^. It is noteworthy that the only false-negative SRS result in our study was in a case of paraganglioma with pathologically confirmed involvement of a contralateral retropharyngeal lymph node approximately 1 cm in size. In addition to size considerations, cellular dedifferentiation in secondary lesions likely accounts for reduced sensitivity of functional imaging methods using receptor-targeting compounds^**(3)**^.

In our sample, the specificity of SRS for paraganglioma detection was 40%, much lower than the 75% reported in one previous study^**(13)**^. Differences in the grading of^111^In-pentetreotide uptake may have contributed to the high rate of false-positive SRS results in the present study. It is also well-established that meningiomas are responsible for false-positive SRS results, because they exhibit^111^In-pentetreotide uptake similar to that of paragangliomas^**(7)**^.

As mentioned, PET studies using^68^Ga-labeled somatostatin analogues currently represent the first-line functional imaging method for the detection of head and neck paragangliomas^**(11)**^. The advantages of PET studies over conventional scintigraphy (including SRS) include better spatial and temporal resolution; the shorter half-life of the PET radionuclides; and the possibility of performing three-dimensional and hybrid imaging^**(2)**^. Nevertheless, as our data show, SRS provides a reasonable option for the detection of head and neck paragangliomas, as well as of residual lesions in patients who have already undergone partial resection, particularly at centers where PET/CT with somatostatin receptor analogues is not available, provided that its limited specificity is taken into account.

The vascular nature of paragangliomas within the head and neck can also be demonstrated by angiography, which optimally depicts tumor perfusion and identifies feeding vessels. Although conventional arteriography is no longer recommended for the diagnosis of head and neck paragangliomas, it is still used for tumor embolization and preoperative occlusion tests, particularly for large lesions of the vagus nerve and skull base^**(11)**^. In the absence of consensus indications, the need for conventional arteriography must be determined on a case-by-case basis. As an alternative, gadolinium-enhanced magnetic resonance angiography, in combination with conventional MRI, has been shown to perform significantly better than conventional MRI alone for the diagnosis of head and neck paragangliomas^**(20)**^.

Paragangliomas may also coexist with other neoplasms. Notably, two patients in our sample had previously been diagnosed with papillary thyroid carcinoma. The association between paraganglioma and papillary thyroid carcinoma has been described^**(21-23)**^. In a study of four patients with paraganglioma and papillary thyroid carcinoma, comprehensive gene analysis was unable to find a single explanation for this association, which may be coincidental or reflect the interaction between different genetic variants^**(24)**^. Paragangliomas and papillary thyroid carcinomas are both associated with mutations in the tyrosine kinase receptor^**(3,25)**^, and paragangliomas may also occur as part of multiple endocrine neoplasm type II syndromes^**(26)**^. Given that these associated tumors may also express somatostatin receptors, SRS may be useful for their detection^**(27,28)**^.
